# Late-Onset Huntington’s Disease in Mexico: A Retrospective Study

**DOI:** 10.7759/cureus.94482

**Published:** 2025-10-13

**Authors:** Adriana Ochoa-Morales, Kerstin Beutelspacher-Fernandez, Aurelio Jara-Prado, Petra Yescas-Gómez, Jorge Luis Guerrero-Camacho, Nelva Jacquelinne Pérez-Pacheco, David Dávila-Ortiz de Montellano, Miguel Ángel Ramírez-García

**Affiliations:** 1 Genetics Department, National Institute of Neurology and Neurosurgery Manuel Velasco Suárez, Mexico City, MEX

**Keywords:** frequency of disease, huntington’s chorea, huntington’s disease (hd), late-onset, mexico

## Abstract

Background

Huntington’s disease (HD) is an autosomal dominant neurodegenerative disorder. It typically presents between the ages of 30 and 40 years. However, a small proportion of cases begin after the age of 60 years, which is referred to as late-onset Huntington’s disease (LoHD). This study aimed to describe the main characteristics of LoHD patients identified at Mexico’s leading neurological center.

Methodology

A retrospective, cross-sectional study was conducted by reviewing records from 1994 to mid-2024. The data collected included the patients’ age, age at onset, diagnostic delay time, family history, molecular outcomes, clinical manifestations at onset, and total motor scores on the Unified Huntington’s Disease Rating Scale-Total Motor Score (UHDRS-TMS).

Results

From 1994 to mid-2024, the National Institute of Neurology and Neurosurgery Manuel Velasco Suárez reported 1,476 individuals with confirmed HD. Of those, 104 (7%) had LoHD, and 56.7% were female. The mean age of onset was 65.1 years. The mean age at molecular confirmation was 71.01 years. The mean diagnostic delay was 6.2 years. The origin of transmission was unknown in 41.3% of cases. Molecular testing revealed an average of 41.4 cytosine-adenine-guanine (CAG) repeats in the expanded allele. Motor symptoms were present at the beginning of the disease in 89% of cases. UHDRS-TMS showed a median score of 29.

Conclusions

LoHD is a rare form of HD with variable manifestations. A common finding in LoHD is the high proportion of patients with no family history of the disease. The lengths of the CAG expanded alleles are typically between 40 and 41 repeats. However, these lengths have also been observed in typical HD presentations. This suggests that further research is needed to identify possible modifiers and understand the pathways that delay disease onset.

## Introduction

Huntington’s disease (HD) is a genetic, neurodegenerative disorder. Involuntary movements, psychiatric disturbances, and cognitive impairment characterize the disorder. HD is inherited in an autosomal dominant pattern and is caused by an expansion of cytosine-adenine-guanine (CAG) trinucleotides in exon 1 of the *HTT* gene located on chromosome 4p16.3 [1]. This expansion encodes a polyglutamine (polyQ) tract at the N-terminal end of the Huntingtin protein. This hinders its degradation, resulting in the selective death of neurons in the basal ganglia and cerebral cortex [2].

The normal range of CAG trinucleotide repeat size is 26 or fewer repeats, with an average of 19 repeats in Mexico. Intermediate alleles range from 27 to 35 repeats. HD is associated with 36 or more CAG repeats. Penetrance is reduced with 36-39 repeats and is complete with 40 or more repeats [3].

There is an inverse correlation between the number of CAG repeats and the age of onset, and the typical/common presentation occurs between the ages of 30 and 40 years [4]. However, juvenile presentations (under 20 years of age) are possible with more than 60 CAG repeats [5]. A small proportion of cases present after the age of 60 years with manifestations similar to the typical presentation. This is termed late-onset Huntington’s disease (LoHD) [6].

This study aimed to present the main characteristics of LoHD patients identified at Mexico’s leading neurological center.

## Materials and methods

A cross-sectional study was conducted via a retrospective review of records from 1994 to mid-2024 at the Genetics Department of the National Institute of Neurology and Neurosurgery Manuel Velasco Suárez (NINNMVS) in Mexico City. The study protocol was reviewed and approved by the scientific and ethics committees (INNN-0423).

Molecular analysis

Until 2015, CAG repeats in the *HTT* gene were evaluated using a radioactively labeled polymerase chain reaction (PCR) with the ^32^P isotope. Since then, triplet-primed PCR has been the standard for assessing the *HTT* expansion.

Sample selection

Cases were identified by evaluating the Genetics Department database. Available clinical records were assessed directly. For individuals whose records had been deleted in accordance with Mexican regulations (NOM-168-SSA1-1998), historical internal records were examined.

The inclusion criteria were as follows: individuals of any sex who presented with clinical manifestations suggestive of HD after the age of 60 years and underwent a molecular test revealing more than 36 CAG repeats in the *HTT* gene. Those presenting with symptoms suggestive of HD after the age of 60 years who did not undergo a molecular test to confirm CAG repeat expansion were excluded.

The following data were obtained from patients’ records: age, age at onset, time of diagnostic delay, family history, molecular outcomes, and clinical manifestations at onset (motor, psychiatric, or cognitive). The total motor score on the Unified Huntington’s Disease Rating Scale-Total Motor Score (UHDRS-TMS) was also obtained.

Statistical analysis

Qualitative variables were described in frequencies and percentages, while quantitative variables were defined as central tendency measures (mean and mode) and dispersion measures (standard deviation and range). We used a Student’s t-test or Mann-Whitney U test to compare quantitative variables between groups, as appropriate. We assessed differences in proportions using the chi-square test. Statistical significance was set at p-values <0.05. All statistical analyses were performed using SPSS software version 30.0 (IBM Corp., Armonk, NY, USA).

## Results

From 1994 to mid-2024, the NINNMVS detected 1,476 individuals with a confirmed HD diagnosis, belonging to 850 families. Of these individuals, 104 (7% of all HD cases) had a presentation of LoHD, and 56.7% (n = 59) were female. The mean age of onset was 65.1 years (SD = 5.27; range = 60 to 90). The mean age at molecular confirmation was 71.01 years (SD = 7.05; range = 60 to 101). The mean diagnosis delay was 6.2 years (SD = 4.93). The transmission was maternal in 42.3% (n = 44) of cases, paternal in 16.3% (n = 17), and unknown in 41.3% (n = 43). Molecular testing revealed a mean of 41.4 CAG repeats in the expanded allele (with a mode of 41 and a range of 36 to 49 CAG repeats), whereas the mean of CAG repeats in the normal allele was 18.3 (with a mode of 15 and a range of 15 to 27).

The initial clinical manifestations were primarily motor (mainly chorea) in 89.4% of cases (n = 93), psychiatric in 78.8% of cases (n = 82), and cognitive in 59.6% of cases (n = 62). The UHDRS-TMS was only available for eight cases at the time of institutional admission, with a median score of 29 (range = 14 to 57). A comparative analysis showed that males exhibited significant cognitive manifestations at disease onset. The rest of the comparisons are shown in Table 1.

**Table 1:** Characteristics of Mexican patients with LoHD. *: t-test; **: Mann-Whitney U test; ***: chi-square test. LoHD: late-onset Huntington’s disease; SD: standard deviation; UHDRS-TMS: Unified Huntington’s Disease Rating Scale Total Motor Score

LoHD characteristics	Females (n = 59)	Males (n = 45)	Total (n = 104)	Statistical test (females vs. males)	P-value
Age of onset, mean ± SD	64.80 ±5.12	65.49 ±5.49	65.1 ±5.27	-0.661*	0.816
LoHD confirmation, mean ± SD	70.45 ±5.78	71.88 ±8.30	71.01 ±7.05	-1.024*	0.386
Diagnosis delay, mean ± SD	5.93 ±4.45	6.56 ±5.53	6.2 ±4.93	-0.629*	0.704
Normal length allele, mean (range)	18.31 (15-26)	16.25 (15-18)	18.3 (15-27)	-0.227*	0.807
Expanded length allele, mean (range)	41.46 (38-48)	41.53 (36-49)	41.4 (36-49)	-0.187*	0.923
UHDRS-TMS, median (range)	37 (28-57)	27.5 (14-57)	29 (14-57)	5.000**	0.486
Manifestations at onset					
Motor, %	88.1 (n = 52)	91.1 (n = 41)	89.4 (n = 93)	0.239***	0.625
Psychiatric, %	74.5 (n = 44)	84.4 (n = 38)	78.8 (n = 82)	1.491***	0.222
Cognitive, %	50.8 (n = 30)	71.1 (n = 32)	59.6 (n = 62)	4.354***	0.037

We explored a Pearson correlation between the age of onset and the expanded allele, but it was not statistically significant (r = -0.04, p = 0.689).

## Discussion

The frequency of LoHD has varied over time depending on the cutoff ages used. This may have led to an overestimation in early reports [6]. However, after setting the age threshold at 60 years, LoHD appears to be present in fewer than 20% of some cohorts described [7,8]. Nevertheless, an Italian cohort exhibited the highest proportion of LoHD cases [9].

In our cohort, the mean age of onset was 65.1 years, with a range of 60 to 90 years, showing the widest range of age in Latin American cohorts [8,10]. Additionally, we found that the frequency of LoHD was 7%. This proportion is similar to that of other Latin American cohorts [8,10,11], but is higher than our population’s previously recognized rate of 4% [12].

In our cohort, we observed a slight difference in the proportion of female LoHD cases. This contrasts with the largest LoHD cohort, which is from the European population and has a more balanced gender distribution [13]. We hypothesized that Mexican female patients with HD would be more interested in being evaluated than males, as we previously observed regarding presymptomatic testing [14]. Additionally, a previous study on typical HD found that women had lower motor and functional scores on the UHDRS, with no difference in age of onset [15]. A high proportion of cognitive impairment has been reported at the onset of symptoms [6]. In our study, however, we found that around 60% of participants exhibited initial cognitive impairment. Interestingly, we also found that this proportion was higher among men.

In our study, the mean number of CAG repeats in the expanded allele was 41.4, which is similar to previously reported findings [6,13,16]. Additionally, we found that a significant proportion (41.3%) had no family history of the pathology, a fact that has been widely recognized [17]. Similarly, allele sizes in late-onset forms may resemble those in the typical presentation. However, the configuration of the repeat and the impact of other genetic factors have been shown to affect the age of HD onset [18,19]. Conversely, there have also been reports of LoHD associated with intermediate alleles [20]. The possible phenotypic effects of this topic are widely debated [21]. Nevertheless, such presentations would fall within the correlational spectrum between repeats and age of onset.

As in previous studies, the most common clinical manifestations were motor symptoms, such as chorea. Psychiatric and cognitive manifestations were less common. Unfortunately, the UHDRS-TMS was only available for eight cases in our study. However, the initial scores were similar to those of a short case series from Argentina (29 vs. 26.9) [10]. Meanwhile, a large European cohort study found that LoHD cases had an initial UHDRS-TMS score consistent with the common/typical age of onset. Nevertheless, higher UHDRS-TMS scores were observed in LoHD patients than in those with a typical age of onset and 41 CAG repeats [13]. However, a study comparing LoHD with the young-onset HD (ages = 20-29) revealed differences in the individual components of the UHDRS-TMS. For example, the LoHD group had higher chorea scores, whereas the young-onset group had higher dystonia and eye movement scores [22].

As observed in this study, LoHD is primarily characterized by motor features, variable progression, and absence of a family history of HD. Further investigation of genetic modifiers in this presentation is necessary to understand the mechanisms that could delay the age of onset and potentially serve as therapeutic targets. Finally, we highlight the following statement from the work of Cornejo-Olivas et al.: “Due to limitations in accessibility, misdiagnosis, and social stigmatization, the actual number of cases of LoHD could be higher.”

Study limitations

Our study has some limitations. Although our LoHD sample is the largest reported among Latin American cohorts, the retrospective design and incomplete medical records (eliminated for compliance) limited our ability to obtain additional clinical data. Furthermore, because there is no cure or preventive treatment, patients often stop attending follow-up appointments.

## Conclusions

LoHD is an infrequent form of presentation of HD, showing variable manifestations and an onset characterized by more motor symptoms than the neuropsychiatric or cognitive features, which are more frequent in the typical presentation. A common finding in LoHD is the high proportion of patients with no family history of HD. Furthermore, the lengths of the CAG expanded alleles may resemble those of the typical presentation. Therefore, further research is needed to identify possible modifiers and understand the pathways that attenuate the phenotype and delay the onset.

## References

[1] Stoker TB, Mason SL, Greenland JC, Holden ST, Santini H, Barker RA (2022). Huntington's disease: diagnosis and management. Pract Neurol.

[2] Waldvogel HJ, Kim EH, Tippett LJ, Vonsattel JP, Faull RL (2015). The neuropathology of Huntington's disease. Curr Top Behav Neurosci.

[3] Caron NS, Wright GEB, Hayden MR (1993). Huntington disease. GeneReviews®.

[4] Gusella JF, MacDonald ME, Lee JM (2014). Genetic modifiers of Huntington's disease. Mov Disord.

[5] Schultz JL, Moser AD, Nopoulos PC (2020). The association between CAG repeat length and age of onset of juvenile-onset Huntington's disease. Brain Sci.

[6] Chaganti SS, McCusker EA, Loy CT (2017). What do we know about late onset Huntington's disease?. J Huntingtons Dis.

[7] Capiluppi E, Romano L, Rebora P (2020). Late-onset Huntington's disease with 40-42 CAG expansion. Neurol Sci.

[8] Cornejo-Olivas MR, Inca-Martinez MA, Espinoza-Huertas K (2015). Clinical and molecular features of late onset Huntington disease in a Peruvian cohort. J Huntingtons Dis.

[9] Volpi E, Terenzi F, Bagnoli S (2021). Late-onset Huntington disease: an Italian cohort. J Clin Neurosci.

[10] Rojas NG, Ziliani JE, Cesarini ME, Etcheverry JL, Da Prat GA, McCusker E, Gatto EM (2019). Late onset Huntington disease: phenotypic and genotypic characteristics of 10 cases in Argentina. J Huntingtons Dis.

[11] Solís-Añez E, Salles PA, Rojas N, Benavides O, Chaná-Cuevas P (2023). Huntington's disease in Chile: epidemiological and genetic aspects. Neuroepidemiology.

[12] Alonso ME, Ochoa A, Boll MC (2009). Clinical and genetic characteristics of Mexican Huntington's disease patients. Mov Disord.

[13] Petracca M, Di Tella S, Solito M (2022). Clinical and genetic characteristics of late-onset Huntington's disease in a large European cohort. Eur J Neurol.

[14] Ochoa-Morales A, Dávila-Ortiz de Montellano DJ, Chávez-Oliveros M (2024). Presymptomatic testing for Huntington's disease in Mexico: 28 years of experience. Arch Med Res.

[15] Zielonka D, Marinus J, Roos RA (2013). The influence of gender on phenotype and disease progression in patients with Huntington's disease. Parkinsonism Relat Disord.

[16] Koutsis G, Karadima G, Kladi A, Panas M (2014). Late-onset Huntington's disease: diagnostic and prognostic considerations. Parkinsonism Relat Disord.

[17] Ramos-Arroyo MA, Moreno S, Valiente A (2005). Incidence and mutation rates of Huntington's disease in Spain: experience of 9 years of direct genetic testing. J Neurol Neurosurg Psychiatry.

[18] (2015). Identification of genetic factors that modify clinical onset of Huntington's disease. Cell.

[19] (2019). CAG repeat not polyglutamine length determines timing of Huntington's disease onset. Cell.

[20] Stoker TB, Holden ST, Barker RA (2021). Late-onset Huntington's disease associated with CAG repeat lengths of 30 and 31. J Neurol.

[21] Ramírez-García MÁ, Dávila-Ortiz de Montellano DJ, Martínez-Ruano L, Ochoa-Morales A, Romero-Hidalgo S, Zenteno JC, Yescas-Gómez P (2022). Clinical and molecular findings of intermediate allele carriers in the HTT gene from the Mexican Mestizo population. Neurodegener Dis.

[22] Anil M, Mason SL, Barker RA (2020). The clinical features and progression of late-onset versus younger-onset in an adult cohort of Huntington's disease patients. J Huntingtons Dis.

